# Combined Inactivation of Pocket Proteins and APC/C^Cdh1^ by Cdk4/6 Controls Recovery from DNA Damage in G1 Phase

**DOI:** 10.3390/cells10030550

**Published:** 2021-03-04

**Authors:** Indra A. Shaltiel, Alba Llopis, Melinda Aprelia, Rob Klompmaker, Apostolos Menegakis, Lenno Krenning, René H. Medema

**Affiliations:** Division of Cell Biology and Cancer Genomics Center, The Netherlands Cancer Institute, Plesmanlaan 121, 1066 CX Amsterdam, The Netherlands; indra.shaltiel@gmail.com (I.A.S.); alballopis@gmail.com (A.L.); maprelia@gmail.com (M.A.); r.klompmaker@nki.nl (R.K.); a.menegakis@nki.nl (A.M.); l.krenning@nki.nl (L.K.)

**Keywords:** cell cycle, checkpoint, CDKs, DNA damage, recovery, G1, CDK4, CDK6

## Abstract

Most Cyclin-dependent kinases (Cdks) are redundant for normal cell division. Here we tested whether these redundancies are maintained during cell cycle recovery after a DNA damage-induced arrest in G1. Using non-transformed RPE-1 cells, we find that while Cdk4 and Cdk6 act redundantly during normal S-phase entry, they both become essential for S-phase entry after DNA damage in G1. We show that this is due to a greater overall dependency for Cdk4/6 activity, rather than to independent functions of either kinase. In addition, we show that inactivation of pocket proteins is sufficient to overcome the inhibitory effects of complete Cdk4/6 inhibition in otherwise unperturbed cells, but that this cannot revert the effects of Cdk4/6 inhibition in DNA damaged cultures. Indeed, we could confirm that, in addition to inactivation of pocket proteins, Cdh1-dependent anaphase-promoting complex/cyclosome (APC/C^Cdh1^) activity needs to be inhibited to promote S-phase entry in damaged cultures. Collectively, our data indicate that DNA damage in G1 creates a unique situation where high levels of Cdk4/6 activity are required to inactivate pocket proteins and APC/C^Cdh1^ to promote the transition from G1 to S phase.

## 1. Introduction

Cell division is a tightly regulated process to ensure proper organismal development and tissue homeostasis. Cyclins and their associated Cyclin-dependent kinases (Cdks) are the key regulators of the cell division cycle and their combined activity is associated with specific cell cycle transitions. More specifically, D-type Cyclins bind to Cdk4 or Cdk6 during the G1-phase of the cell cycle, triggering the induction of expression of Cyclin E, which in turn causes activation of Cdk2 that binds to the E-type Cyclins at the onset of DNA replication. Cdk2-associated A-type Cyclins subsequently enable progression through S- and G2-phases, and ultimately Cdk1-associated B-type Cyclins promote the transition through mitosis [1]. Collectively, Cyclin-Cdk complexes coordinate transcriptional programs, the activity of cell cycle-dependent ubiquitin-ligases, replication origin firing, and structural reorganization of organelles required for cell division [2,3].

To maintain genomic integrity, the cell cycle machinery must be responsive to internal and external cues that determine when a cell can commit to the next irreversible transition in the cell cycle. Initiation of DNA replication in the presence of damaged DNA can have severe deleterious consequences, as progression of the replication fork will turn single-strand breaks into double-strand breaks, while replication of modified residues may give rise to the accumulation of mutations and damaged bases may interfere with replication in general [4,5,6]. Additionally, failure to prevent mitotic entry before damage has been repaired can lead to irreversible damage to the genome [7]. To deal with these deleterious effects, cells are equipped with cell cycle checkpoints that are activated in response to DNA damage and can prevent cell cycle progression and allow for timely repair [8]. For example, DNA damage in G1 evokes a DNA damage checkpoint response that inhibits Cdk2 activity and prevents initiation of DNA replication [9]. This requires the kinase ataxia telangiectasia mutated (ATM) to activate downstream kinases p38 and Chk2 [10,11,12] and promotes stabilization of the tumor suppressor p53 [13] resulting in the induction of its transcriptional target, the Cdk inhibitor p21 [14]. This cascade of events results in an effective shutdown of Cdk activity and causes cells to arrest in G1. In a prolonged G1 arrest, the Cdk inhibitor p27 is additionally induced [15]. Both p21 and p27 bind to Cyclin-Cdk complexes and inhibit their activities, which in G1 are required for the phosphorylation and inactivation of pocket proteins pRb, p107 and p130 [16]. The resultant non- and hypophosphorylated pocket proteins bind and inhibit the family of E2F transcription factors, preventing them from activating their target genes, that are collectively required for S-phase entry [17].

Despite the well-organized and evolutionarily conserved action of the various Cyclin-Cdk complexes, there is ample redundancy within the cell cycle network. Mice in which *cdk2* and *cdk4* are genetically deleted are viable, and even mouse embryos deficient for *cdk2*, *cdk3*, *cdk4*, and *cdk6* develop to mid-gestation [18,19], indicating that most Cdks are redundant for cell cycle progression in general, and S-phase entry in particular. Similar redundancies have been observed between Cyclins, with all D-Cyclins and E-Cyclins being dispensable up to mid-gestation in mice [20,21,22]. Finally, deletion of all activator E2F transcription factors can allow for normal cell cycle progression under certain conditions [23,24,25].

This ubiquitous redundancy within the cell cycle machinery may reflect requirements for selected Cdks in particular cellular situations, such as during the cell cycle restart following a DNA damage-induced arrest. Here, we have tested the role of different Cyclin/Cdk subunits in cells during recovery from a DNA damage-induced arrest in G1.

## 2. Materials and Methods

### 2.1. Cell Lines

hTert-immortalized retinal pigment epithelium (RPE) and derived cell lines were maintained in DMEM/F12 (Gibco) supplemented with ultraglutamine, penicillin/streptomycin, and 6% fetal bovine serum. RPE-FUCCI cells have been described before [26]. RPE-FLAG-Cdk4(NT), RPE-FLAG-Cdk4(NT/KD), and RPE-FLAG-Cdk6 were obtained as polyclonal cell lines after retroviral transduction with the corresponding pBABE constructs and puromycin selection. RPE-1 with doxycycline-inducible expression of E7 was generated by retroviral transduction of RPE-1 cells stably expressing an ecotropic receptor and the Retro-X Tet-On Advanced Transactivator (Clontech) with pRetroX-tight-puro-E7 followed by puromycin selection.

### 2.2. Constructs

Cdk4 cDNA (Origene) was subjected to site directed mutagenesis using 5′-ctgaccgggagatcaaagtaacactggtctttgagcatgtagacc-3′ and complementary primers to generate a construct insensitive to Dharmacon siRNA#1 (non-targetable; NT). Kinase-dead Cdk4 was generated by additional site-directed mutagenesis using 5′-gaacagtcaagctggctaactttggcctggc-3′ and complementary primers yielding Cdk4 D158N. pBABE-FLAG-Cdk4(NT) and pBABE-FLAG-Cdk4(NT/KD) were obtained by cloning the PCR products of 5′-gatGGATCCatggactacaaagacgatgacgacaagGCTACCTCTCGATATGAGCCAGTG-3′ and 5′-gcataGAATTCtcactccggattaccttcatccttatg-3′ primers with the introduced BamHI and EcoRI restriction sites into corresponding sites of pBABE-puro. To obtain pBABE-FLAG-Cdk6, the Cdk6 CDS was amplified from RPE-1 cDNA and was further amplified with 5′-gatGGATCCatggactacaaagacgatgacgacaagGAGAAGGACGGCCTGTGCCGCG-3′ and 5′-gcataGAATTCtcaggctgtattcagctccgagg-3′ primers to introduce the FLAG-tag and restriction sites for BamHI and EcoRI. pBABE-E7 was a gift of René Bernards. pRetroX-tight-pur-E7 was obtained by PCR-mediated introduction of EcoRI and BamHI restriction sites and ligation of the product into corresponding sites of the vector.

### 2.3. Antibodies and Reagents

Antibodies used in this study are the following: antibodies directed against Cdk4 (C-22, Cdk6 (C-21), p107, pRb pS807/811, p21, p53 (DO-1), p130 (C-20), beta actin (Santa Cruz Biotechnology, Santa Cruz, CA, USA), H2AX pS139 (Millipore, Burlington, MA, USA), alpha tubulin, FLAG (Sigma Aldrich, Saint Louis, MO, USA), p27, pRb, (BD Biosciences, San Jose, CA, USA). The following reagents were used: doxycycline (1 μg/mL; Sigma Aldrich, Saint Louis, MO, USA), Nutlin-3 (5 μM; Sigma Aldrich), S-trityl-L-Cysteine (10 μM; Sigma Aldrich), PD0332991 (100 nM or 500 nM), p38 ([SB202190; 3 μM; Millipore) and Chk2 (Chk2 inhibitor II; 10 μM; Sigma Aldrich), RO-3306 (10 μM; Calbiochem, San Diego, CA, USA), SNS-032 (5 μM; Selleckchem, Houtston, TX, USA).

### 2.4. siRNA Transfections and Automated Microscopy

siRNAs were purchased as ON-TARGET*plus* pools from Dharmacon (now Horizon Discovery, Lafayette, CO, USA). We used GAPDH or luciferase siRNA as control siRNA. After serum withdrawal, we transfected cells with 20 nM pooled siRNA using RNAiMAX transfection reagent (Invitrogen, Carlsbad, CA, USA). We irradiated the cells six hours after serum restimulation with 4 Gy from a shielded Cs-137 source and supplemented medium with 5′-ethynyl-2′-deoxyuridine (EdU; 10 μM; Invitrogen). For G1 checkpoint recovery, Chk2 inhibitor II and SB202190 were added 16 hrs after irradiation and cells were allowed to recover in the continuous presence of EdU for an additional 24 h. Either 24 h after mock irradiation or checkpoint silencing, we fixed cells in 3% formaldehyde in PBS and stained for EdU incorporation with click chemistry (100 mM Tris pH 8.5, 100 mM ascorbic acid, 1 μM AlexaFluor 488-azide (Invitrogen), 1 mM CuSO_4_) as described [27]. Nuclei were counterstained with 4′,6-diamidino-2-phenylindole (DAPI; 1 μg mL^−1^; Sigma Aldrich) and imaged in a Cellomics Arrayscan automated fluorescence microscope with a 20× (NA 0.4) Zeiss Axiovert 200M objective. Each condition was performed in duplicate or triplicate, and at least 400 cells were imaged per well. EdU-positive cells were identified with a fixed threshold of the average fluorescence intensity in the nucleus over the immediate background in a ring around the nucleus.

### 2.5. Time-Lapse Microscopy

Asynchronously proliferating RPE-FUCCI cells were transfected with indicated siRNAs using RNAiMAX transfection reagent (Invitrogen) in Lab-Tek II (Thermo Scientific, Waltham, MA, USA) chambers. Time lapse was set up 24 to 48 h after transfection, immediately after (mock) irradiation from a caesium-137 source and replacement of medium with pre-warmed Leibovitz’s L-15 medium containing all supplements. For spontaneous recovery, images were acquired with a CoolSNAP-HQ2 camera and an Olympus 10× (NA 0.40) U-Plan S-Apo objective with Quad-mCherry polychroic mirror and GFP/mCherry emission filters at 15-min intervals on a Deltavision system (Applied Precision). >60 G1 (clear/red fluorescent) and >60 S/G2 (green fluorescent) cells were followed per condition per experiment from the first frame of the experiment until the last frame or mitosis.

### 2.6. Immunofluorescence and Automated Image Analysis

Cells grown on glass cover slips were synchronized and transfected with siRNAs as before. At indicated time points after irradiation and inhibitor treatment, we fixed cells in PBS-buffered 3.7% formaldehyde, permeabilized with −20 °C methanol and blocked aspecific binding with TBS containing 4% BSA and 0.1% Tween-20 prior to antibody incubation for immunofluorescence. Images were collected with an Olympus U-Plan S-Apo 20× (NA 0.75), or an Olympus 40× (NA 0.85) IMT2 objective, Quad/mCherry polychroic mirror and Alexa Fluor filter sets on a Deltavision (Applied Precision) system and mean fluorescence intensity per nucleus was determined using ImageJ software (http://rsb.info.nih.gov/ij/, accessed on 24 February 2021).

For H2AX pS139 foci analysis, we made use of previously developed automated image analysis software [28]. In short, this software segments nuclei based on thresholding the DAPI signal. Foci background was substracted using a Difference-of-Gaussians filter, applied on maximum intensity projections. For individual nuclei, foci were identified as regions of adjacent pixels that follow these criteria: (i) the intensity value exceeds the background signal by a few fold (typically 2–4×) of the standard deviation median of the background of all nuclei in the image, and is higher than a user-defined absolute minimum signal; (ii) the area is larger than a user defined area.

### 2.7. Western Blot

For Western Blot, cells were lysed in Laemmli sample buffer, protein was separated by SDS-PAGE and transferred to a nitrocellulose membrane (Whatman, Maidstone, UK), stained with the indicated antibodies and visualized by chemiluminescence (GE Healthcare, Chicago, IL, USA).

## 3. Results

### 3.1. Recovery from a DNA Damage-Induced G1 Arrest Requires Cdk4 and Cdk6

**Figure 1:**
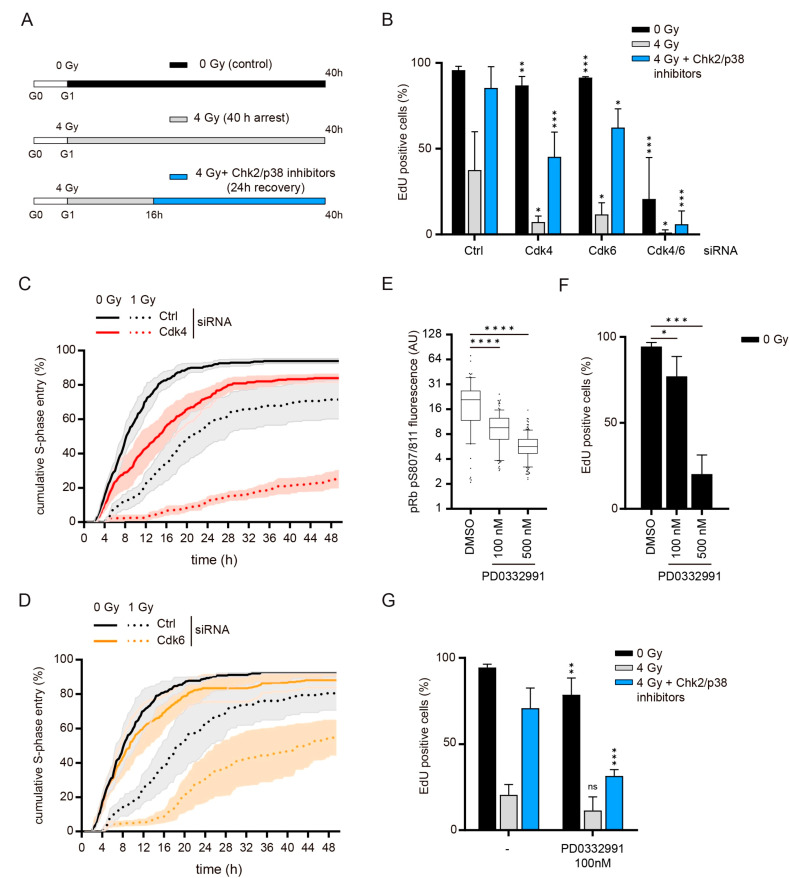
Cdk4 and Cdk6 are required to promote G1 checkpoint recovery: (**A**) G1 recovery assay. RPE-1 cells were synchronized in G0 by contact inhibition and 48 h of serum starvation. After 4 to 6 h of serum restimulation and EdU addition (G1), cells were mock-treated or irradiated with 4 Gy in G1. To study recovery from DNA damage, cells were left untreated or Chk2 and p38 inhibitors were added 16 h after irradiation for additional 24 h. (**B**) G1-synchronized RPE-1 cells were depleted of Cdk4 or Cdk6 by siRNA-transfection during serum starvation and treated as in (**A**). Percentage of cells entering S-phase was assessed by EdU incorporation. Depicted are the means and SD of four independent experiments. Significance was calculated by comparing different knock-downs to GAPDH-depleted control cells of similar irradiation regimen (e.g., Cdk4-depleted 4 Gy irradiated samples are compared to control-depleted 4 Gy irradiated samples). (**C**,**D**) Asynchronously proliferating RPE-FUCCI cells were subjected to time-lapse fluorescence microscopy 24 to 48 h after siRNA transfection. Cells in G1 at the start of the experiment were followed to determine cumulative S-phase entry. For irradiated conditions, entry of cells beyond the restriction point was excluded (i.e., S-phase entry in the first four hours after irradiation). Depicted are the means and SEM of four (**C**) and three (**D**) independent experiments. (**E**) G1 synchronized RPE-1 cells were treated with the indicated PD0332991 concentrations for 16 h and subsequently fixed for immunofluorescent detection of S807/811 phosphorylated pRb. Depicted is one representative experiment. (**F**). G1 synchronized RPE-1 cells were treated with the indicated concentrations of PD0332991 for 40 h, and S-phase entry analyzed by EdU staining. Depicted are the means and SD of three independent experiments. (**G**) G1 synchronized RPE-1 cells were treated as in (**A**). PD0332991 was added before mock or 4 Gy irradiation in G1 when indicated. Depicted are the means and SD of four independent experiments. Significance was calculated by comparing drug treated samples to control cells of similar irradiation regimen (e.g., 4 Gy irradiated and drug treated samples were compared to 4 Gy irradiated cells that were not treated with PD0332991). For all panels, significance was calculated using a one-sided unpaired *t*-test. * *p* < 0.05, ** *p* < 0.01, *** *p* < 0.005, **** *p* < 0.0001, ns = not significant.

We have previously shown that the DNA damage response in G1 is lost in commonly used p53-proficient cancer cell lines [29], but is retained in immortalized non-transformed human retinal pigment epithelial cells (RPE-1) [26]. We and others showed that the DNA damage checkpoint in G1 is maintained by p38 and Chk2 kinases in non-transformed cells [26,30,31]. When inhibitors of these kinases are added to G1-arrested RPE-1 cells (16 h after irradiation with 4 Gy in G1), we can induce S-phase entry in approximately 80% of the population in 24 h (Figure 1A,B). We used this setup to determine whether there are particular components of the cell cycle machinery that are dispensable for normal G1/S progression, but required for recovery from a DNA damage-induced arrest. To this end, we transfected RPE-1 cells with siRNA pools targeting the various cell cycle-implicated Cyclins, Cdks and Cdc25 phosphatases during serum starvation, and measured S-phase entry by EdU incorporation upon serum stimulation (Figure 1A,B and Figure S1A).

After restimulation, 90% of control, GAPDH-depleted cells progressed to S-phase (Figure S1A). In contrast, single depletion of Cdk2, Cyclin A2, Cyclin D1, or combined depletion of Cdk4 and Cdk6 (Cdk4/6), strongly reduced S-phase entry of non-irradiated RPE-1 cells, suggesting that these proteins are essential for normal S-phase entry.

Interestingly, we found that S-phase entry after DNA damage was specifically impaired after siRNA-mediated knockdown of Cdk4 or Cdk6 alone (Figure 1B and Figure S1A). Single depletion of Cdk4 or Cdk6 allowed normal S-phase entry of non-irradiated cells, but resulted in a 40 to 60% reduction in the population recovering from irradiation (Figure 1B). To confirm that this dependence on Cdk4 and Cdk6 also exists during spontaneous recovery from DNA damage, we analyzed S-phase entry after irradiation (1 Gy) in asynchronously proliferating RPE-1 cells expressing live cell cycle fluorescence markers (RPE-FUCCI) [32]. Single Cdk4 or Cdk6 depletion did not affect S-phase entry of non-irradiated cells (Figure 1C,D), but combined Cdk4/6 knockdown prevented normal S-phase progression in asynchronously growing cells (Figure S1B). Consistent with the reduced recovery observed after p38 and Chk2 inhibition, spontaneous recovery of G1 irradiated cells was impaired after Cdk4- or Cdk6-depletion. Only 25% of the Cdk4-depleted and 55% of the Cdk6-depleted G1 cells progressed to S-phase within 48 h after 1 Gy, compared to 70 to 80% of the siRNA control G1 cells (Figure 1C,D). We detected no major effects of Cdk4 or Cdk6 depletion on cell cycle progression of G2 cells either with or without irradiation (Figure S1C). Thus, while only one of two canonical G1 Cdks, Cdk4 or Cdk6, is enough for normal S-phase entry in RPE-1 cells, both of them become critical for S-phase entry after DNA damage.

Since both Cdk4 and Cdk6 are required for recovery in G1, we wondered if this was due to independent activities of both kinases, or simply a reflection of a higher dependency of G1-arrested cells on overall Cdk4/6 activity. To this end we tested if the recovery defect induced by Cdk4-depletion, could only be overcome by restoring Cdk4 expression, or if it could also be rescued by increasing Cdk6 activity. Ectopic expression of an RNAi-insensitive Cdk4 or Cdk6 both fully restored G1 recovery in Cdk4-depleted cells (Figure S1D), suggesting that DNA damaged cells are critically dependent on the overall Cdk4/6 activity. Indeed, re-introduction of a kinase-dead mutant of Cdk4 (Cdk4-KD) in Cdk4-depleted RPE-1 cells did not restore S-phase entry after DNA damage (Figure S1D), showing that it is the overall level of Cdk4/6 kinase activity that determines if a cell can recover from the G1 arrest induced by DNA damage. Additionally, this observation excludes the possibility that Cdk4/6 promote recovery by sequestering away the Cdk inhibitor proteins p21 and p27 [33].

Next, we assessed if we could confirm the greater dependency on Cdk4/6 activity in DNA-damaged cells using chemical inactivation of Cdk4 and Cdk6. To this end, we added different concentrations of PD0332991, a Cdk4/6 inhibitor, to G1-synchronized cells. PD0332991 caused a dose-dependent reduction in pRb phosphorylation (Figure 1E). We found that a 500 nM concentration of PD0332991 prevented normal S-phase entry in 80% of the RPE-1 cells (Figure 1F). To exclude that this concentration of PD0332991 inhibited other cell cycle Cdks we analyzed the effect of 500 nM PF0332991 on Cdk2 and Cdk1 activity using a previously reported Cdk2 activity sensor [34] and by quantifying mitotic entry of cells with active Cdk2, respectively. We found that 500 nM PD0332991 did not directly inhibit Cdk2 (Figure S1E). Additionally, PD0332991 did inhibit Cdk1, as it did not prevent cells from entering into mitosis (Figure S1F). Over the course of 5 h PD0332991 reduced the average Cdk2 activity (Figure S1E), likely due to an accumulation of cells with inactive Cdk2 in G1-phase. Thus, we conclude that the inhibition of S-phase entry by PD0332991 was caused by inhibition of Cdk4 and Cdk6. In contrast, a 100 nM concentration of PD0332991 hardly affected normal S-phase entry but severely impaired DNA damage recovery (Figure 1F,G). Thus, partial inhibition of Cdk4/6 with PD0332991 allows normal S-phase progression but blocks recovery from DNA damage, mimicking the phenotype observed after single Cdk4 or Cdk6 depletion. Our results show that irradiated G1 cells become critically dependent on overall Cdk4/6 activity to re-enter the cell cycle, and suggest that the G1 to S transition after DNA damage requires a higher level of Cdk4/6 activity than normal S-phase entry.

### 3.2. Cdk4/6 Activity Is Not Required during the Arrest

**Figure 2:**
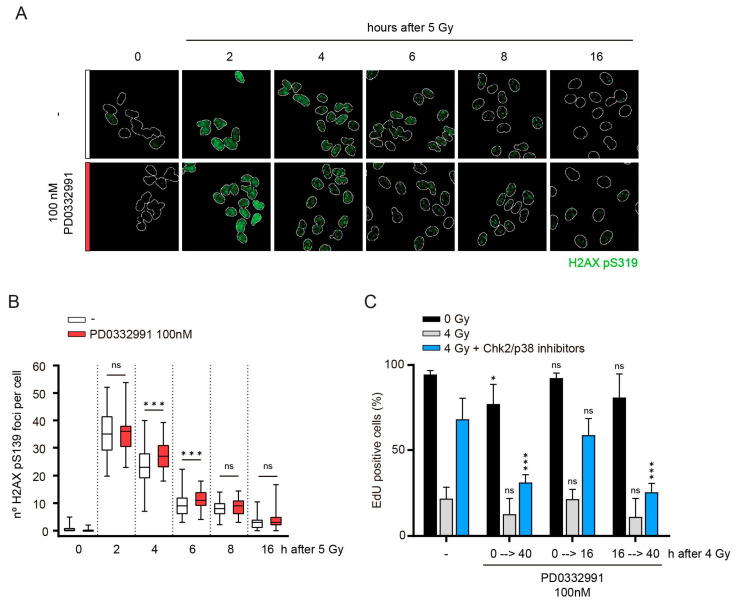
Cdk4/6 activity is not required during a G1 arrest: (**A**) RPE-1 cells synchronized in G1 were irradiated and stained for H2AX pS139. White outlines demarcate nuclei based on DAPI counterstains. (**B**) Quantification of H2AX pS139 foci in (**A**) using previously developed image analysis software (Warmerdam et al., 2019). Whiskers represent 5–95% of data points. At least 40 cells were quantified per condition. (**C**) Cells were treated as in 1A. Two different doses of Cdk4/6 inhibitor PD0332991 were applied either throughout the first 16 h of the experiment (0→16), throughout the experiment (0→40) or from the moment of Chk2 and p38 inhibitor addition (16→40). Depicted are means and SD from three independent experiments. Significance was calculated by comparing different drug regimens to control cells of similar irradiation regimen (e.g., 4 Gy irradiated and PD0332991-treated samples (0→40) were compared to 4 Gy irradiated cells that were not treated with PD0332991). For all panels, significance was calculated using a one-sided unpaired *t*-test. * *p* < 0.05, *** *p* < 0.005, ns = not significant.

Since the recovery defect in Cdk4- and Cdk6-depleted cells is seen after artificial silencing of the DNA damage checkpoint with small molecule inhibitors, we expected the Cdk4/6 requirement to be independent of DNA repair and checkpoint silencing. Indeed, the resolution of foci of S139-phosphorylated histone variant H2AX (γH2AX), a marker of sites of DNA damage, occurred largely normal in the presence of 100 nM PD0332991, and there was no detectable difference in DNA damage foci at 8 or 16 h post irradiation (Figure 2A,B).

To study when exactly Cdk4 and Cdk6 are required to promote recovery, we inhibited Cdk4/6 at different stages of the DNA damage response. As shown above (Figure 1G), addition of 100 nM PD0332991 immediately before irradiation (from 0 h to the end of the experiment, 40 h after 4 Gy) prevented G1 recovery (Figure 2C). When Cdk4/6 activity was inhibited only during the first sixteen hours of the arrest, recovery was indistinguishable from non-treated RPE-1 cells (Figure 2C). In contrast, addition of 100 nM PD0332991 together with Chk2 and p38 inhibitors (from 16 h to 40 h after 4 Gy) prevented S-phase entry of the irradiated cells. These data indicate that Cdk4/6 kinase activity is not essential for DNA damage repair, but only required once the (majority of) breaks have been resolved and the cell cycle machinery needs to be re-activated.

### 3.3. Cdk4/6 Activity Is Counteracted by p21 but not by p27 after DNA Damage

**Figure 3:**
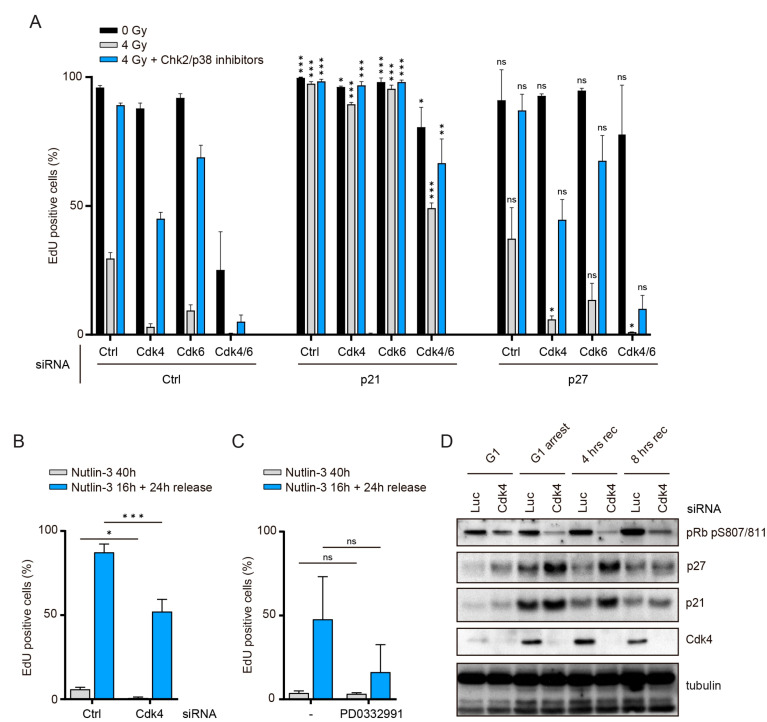
Regulation of p21 and p27 Cdk inhibitors in G1 recovery: (**A**) RPE-1 cells were transfected with the indicated siRNA during serum starvation and G1 recovery was assessed as in 1A. Depicted are the means and SD of two independent experiments. Significance was calculated by comparing the knock-down of different Cyclin-dependent kinases (Cdks) in combination with either p21 or p27 knockdown to the different Cdk knock-downs combined with a control siRNA, treated with a similar irradiation regimen (e.g., p21+Cdk4-depleted 4 Gy irradiated samples are compared to control+Cdk4-depleted 4 Gy irradiated samples). (**B**) RPE-1 cells were transfected with Cdk4 or control siRNA during serum starvation and treated in G1 with 5 μM Nutlin-3 for 16 h, when the inhibitor was either maintained or washed out (release). Cells were harvested 24 h after Nutlin-3 washout. S-phase entry was determined by EdU incorporation. Depicted are means and SD from three independent experiments. (**C**) Cells were treated as in (**B**). PD0332991 was added together with Nutlin-3 and during the release, where indicated. S-phase entry was determined by EdU incorporation. Depicted are the means and SD from 4–6 replicates from two independent experiments. (**D**) Cells treated as in (**A**) were collected at indicated time points for Western Blot. For all panels, significance was calculated using a one-sided unpaired *t*-test. * *p* < 0.05, ** *p* < 0.01, *** *p* < 0.005, ns = not significant.

We next wondered what constituted the difference between a G1-arrested state due to serum starvation and after irradiation. Cdk4/6 activity is low both after serum starvation or exposure to ionizing radiation, yet in the irradiated cells this is due to enhanced expression of p27 and p21 rather than a lack of growth factor signals [14,35]. Thus, we first assessed whether the recovery defect of Cdk4- or Cdk6-depleted cells was due to a failure to overcome inhibition by p27 with co-depletion experiments. Knockdown of p27 did not alter S-phase entry in non-irradiated, irradiated or recovery conditions in cells with normal Cdk4 and Cdk6 levels (Figure 3A). Notably, p27 depletion restored normal S-phase entry in Cdk4/6 double depleted cells, consistent with a role for Cdk4/6 during normal cell cycle progression in overcoming the inhibitory action of p27 [33]. In contrast, depletion of p27 had no notable impact on cell cycle reentry after DNA damage in Cdk4- and Cdk6-depleted cells (Figure 3A), ruling out a critical role for p27 in creating the observed Cdk4 and Cdk6 dependence.

We next addressed whether Cdk4/6 are required to overcome p21 during recovery from irradiation. However, since p21-depleted cells fail to arrest in G1 in response to DNA damage (Figure 3A) [14], co-depletion experiments could not address the role of p21 during recovery. As an alternative approach to address the interplay between Cdk4/6 and p21, we tested whether p53-mediated p21 induction is sufficient to precipitate a dependency on Cdk4/6 activity for further cell cycle progression. To this end, we stabilized p53 and induced p21 without inducing DNA damage using Nutlin-3, a small molecule inhibitor of Mdm2 (Figure 3B,C). After washout of Nutlin-3, Cdk4-depleted cells, or cells where Cdk4/6 activity was partially inhibited, performed poorer in cell cycle progression than control cells (Figure 3B,C). Thus, Cdk4/6 activity is required to fully counteract a p53-dependent arrest. Finally, we analyzed p21 and p27 levels during recovery from DNA damage in Cdk4-depleted cells. p21 and p27 protein levels were higher in Cdk4-depleted cells in comparison with control depleted cells 4 h after induction of recovery (Chk2 and p38 inhibitors addition), but reduced to a similar extent 8 h after induction of recovery (Figure 3D). Overall, these data indicate that the requirement for Cdk4 and Cdk6 in recovery from a DNA damage-induced arrest in G1 does not stem from a direct role in the inactivation of p27 or p21, neither to sequester them away from Cdk2, nor to promote their turnover. However, some of the dependency on Cdk4 and Cdk6 can be recapitulated by p53 stabilization alone, showing that Cdk4/6 activity is required to completely revert to a p53-dependent G1 arrest.

### 3.4. The Role of Cdk4/6 during Recovery Surpasses the Inactivation of Pocket Proteins

**Figure 4:**
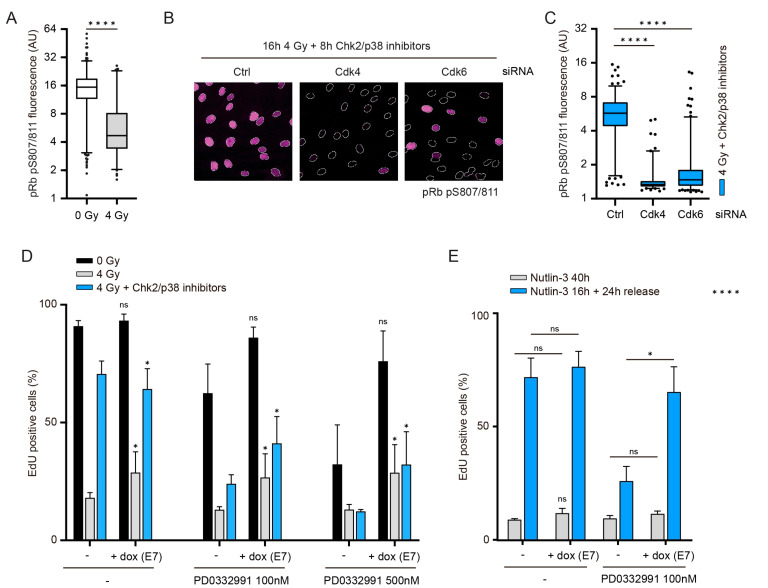
Cdk4/6 are required for more than pocket protein inactivation: (**A**) RPE-1 cells were synchronized in G1 and (mock) irradiated. Cells were fixed 8 h later for immunofluorescent detection of S807/811-phosphorylated pRb. (**B**) RPE-1 cells were transfected with siRNAs during serum starvation and irradiated (4 Gy) in G1. 16 h after irradiation, Chk2 and p38 inhibitors were added and cells were collected 8 h afterwards for immunofluorescent detection of S807/811-phosphorylated pRb. White outlines demarcate nuclei based on DAPI counterstaining. Nuclear fluorescence intensities were quantified in (**C**). At least 100 cells were quantified per condition. (**D**) RPE-E7 cells were synchronized in G1 and mock-treated or irradiated (4 Gy). 16 h after irradiation, doxycycline, PD0332991, and Chk2/p38 inhibitors were added where indicated for additional 24 h. S-phase entry was detected by EdU incorporation. Depicted are the means and SD of three independent experiments. Significance was calculated by comparing cells treated with doxycycline to cells treated without doxycycline, that were otherwise treated the same (e.g., 4 Gy irradiated cells treated with 100 nM PD03329991 and doxycycline were compared to 4 Gy irradiated cells treated with 100 nM PD0332991 without doxycycline). (**E**) Nutlin-3 was added to G1-synchronized RPE-E7 cells for 16 h, when it was either maintained or washed out (release). PD0332991 and doxycycline were added during the release, where indicated. S-phase entry was determined by EdU incorporation. Depicted are the means and SD from 4–6 replicates from two independent experiments. For all panels, significance was calculated using a one-sided unpaired *t*-test. * *p* < 0.05, **** *p* < 0.0001, ns = not significant.

We next wanted to know what the downstream targets of Cdk4/6 might be that control cell cycle re-entry after DNA damage. The pocket proteins pRb (retinoblastoma protein), p107 and p130 are the canonical substrates of Cdk4 and Cdk6, and their functions are inhibited by phosphorylation [36,37]. We observed that pRb phosphorylation is low after irradiation (Figure 4A), but phosphorylation is regained after checkpoint silencing in a Cdk4- and Cdk6-dependent manner (Figure 3D, Figure 4B,C and Figure S2A). To examine whether impaired phosphorylation of pocket proteins by Cdk4/6 inhibition causes the irreversible arrest in G1 after irradiation, we first inactivated the three pocket proteins by co-depletion together with Cdk4 or Cdk6 (Figure S2B,C). Co-depletion of p107 and p130 with Cdk4 or Cdk6 failed to improve cell cycle reentry of irradiated cells, suggesting that the lack of p107 and p130 phosphorylation is not the cause of the defect observed in Cdk4- or Cdk6-depleted cells. Knockdown of pRb restored S-phase entry in Cdk4/6 double-depleted cells, showing that the requirement for Cdk4 and Cdk6 during a normal G1 is to inactivate pRb. Surprisingly, co-depletion of pRb with Cdk4 or Cdk6 did not rescue cell cycle reentry after irradiation (Figure S2B). It should be noted that we find that pRb-depleted RPE-1 cells can be arrested in G1, whereas genetic Rb-null embryonic fibroblasts have been described to lack a G1 checkpoint arrest in response to irradiation [38], challenging the quality of the knockdown in our experiments (Figure S2C). Nonetheless, the differential effect of pRb-depletion on unperturbed versus DNA-damaged G1 cells clearly shows that Cdk4/6 are required for more than just inactivation of pRb.

As an independent approach to inactivate pocket proteins, we made use of the viral E7 oncoprotein, known to bind and inactivate all pocket proteins [39,40]. We generated an RPE-1 cell line in which E7 transcription was under the control of a doxycycline-responsive promoter (RPE-E7). This setup allowed us to specifically test whether Cdk4 and Cdk6 are primarily required to shut down pocket proteins after an established arrest. While E7 was expressed at low levels in uninduced RPE-E7 cells, the addition of doxycycline severely increased E7 expression (Figure S3A). Despite their low level of E7 expression, uninduced RPE-E7 cells showed a similar DNA damage induced G1 arrest compared to wild type RPE-1 cells (compare Figure 4D to Figure 1C,G, Figure 2C and Figure 3A). Induction of E7 in G1 restored S-phase entry when Cdk4/6 were completely inhibited, validating the system and confirming that pocket proteins are the main targets of Cdk4/6 in a normal G1 (Figure 4D). In contrast, inactivation of pocket proteins in DNA damage-arrested G1 cells could not promote full recovery when Cdk4/6 activity was inhibited (Figure 4D). This shows that Cdk4/6 have (an)other substrate(s), besides the pocket proteins, that need to be (in)activated to promote recovery. Finally, we addressed the role of pocket proteins in recovery from a Nutlin-3-induced arrest. Interestingly, induction of E7 after Nutlin-3 washout completely restored S-phase entry in Cdk4/6-inhibited cells (Figure 4E), indicating that the other target of Cdk4/6 in recovery is not a direct target of p53. Taken together, these data show that, although phosphorylation of pRb is regulated by Cdk4/6 after irradiation, inactivation of pocket proteins is not sufficient to completely rescue G1 recovery after irradiation in the absence of Cdk4 or Cdk6. In contrast, Cdk4/6′s requirement for recovery after p53 and p21 induction can be counteracted by the inactivation of pocket proteins.

### 3.5. Combined Inactivation of Pocket Proteins and Cdh1 Enables Cdk4/6-Independent Recovery

Cdh1 is the coactivator subunit of the APC/C ubiquitin ligase during G1, and it has been recently shown that inactivation of pRb and Cdh1 synergize to overcome Cdk4/6 inhibition in normal, non-DNA damage conditions [41]. We therefore tested whether inactivation of Cdh1 could overcome the dependence on Cdk4/6 for recovery after DNA damage.

**Figure 5:**
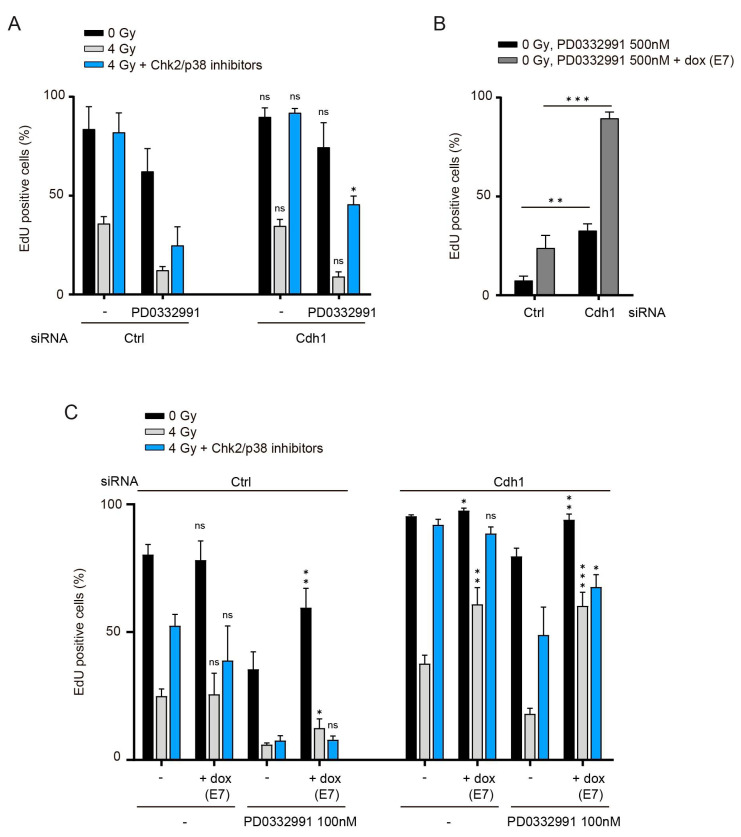
Cdh1 depletion and pocket proteins inactivation restore G1 recovery in the absence of Cdk4/6 activity: (**A**) RPE-1 cells were transfected with the indicated siRNA during serum starvation and G1 recovery was assessed as in 1A. PD0332991 100 nM was added 16 h after irradiation when indicated. Depicted are the means and SD from three independent experiments. Significance was calculated by comparing control or Cdh1-depleted cells that where otherwise treated similarly (e.g., Cdh1-depleted 4 Gy irradiated cells that were treated with PD0332991 were compared to control-depleted 4 Gy irradiated cells that were treated with PD0332991). (**B**) RPE-E7 cells were transfected with the indicated siRNA during serum starvation. PD0332991 500 nM was added in G1 for 40 h, together with doxycycline, when indicated. Depicted are the means and SD of two independent experiments. (**C**) RPE-E7 cells were transfected with the indicated siRNA during serum starvation and G1 recovery was assessed as in 1A. PD0332991 and doxycycline were added 16 h after irradiation where indicated. Depicted are the means and SD of three independent experiments. Significance was calculated by comparing cells treated or not with doxycycline, that were otherwise treated similarly (e.g., Cdh1-depleted cells that were 4 Gy irradiated and subsequently treated with Chk2/p38 inhibitors, PD0332991 and doxycycline were compared to the same cells without doxycycline). For all panels, significance was calculated using a one-sided unpaired *t*-test. * *p* < 0.05, ** *p* < 0.01, *** *p* < 0.005, ns = not significant.

First, we analyzed S-phase entry after irradiation in Cdh1-depleted cells with or without Cdk4/6 inhibition. Transfection of Cdh1-targeting siRNAs resulted in a stark decrease of Cdh1 expression (Figure S3B). Cdh1 depletion did not alter the DNA damage arrest or recovery in cells with normal Cdk4/6 activity levels. However, Cdh1 depletion partially restored the defect in S-phase entry after DNA damage caused by Cdk4/6 inhibition (Figure 5A). Next, we asked whether we could completely rescue the defect of G1 recovery caused by Cdk4/6 inhibition with the combined inactivation of pocket proteins and Cdh1. We made use of the RPE-E7 inducible cell line and analyzed S-phase entry in the presence of Cdk4/6 inhibition. Defective S-phase entry caused by inhibition of Cdk4/6 activity was partially alleviated by Cdh1 depletion, consistent with previous observations [41], and completely rescued by combined inactivation of pocket proteins and Cdh1 (Figure 5B). However, more importantly, Cdh1 depletion and E7 induction also restored S-phase entry after DNA damage in the presence of Cdk4/6 inhibition (Figure 5C). Remarkably, combined inactivation of pocket proteins and Cdh1 was sufficient to allow almost complete recovery even in the absence of p38 and Chk2 inhibitors. Thus, our data show that pocket proteins and Cdh1 act together to maintain a G1 arrest, and indicate that Cdk4/6 activity is required to counteract them during recovery from DNA damage in G1.

## 4. Discussion

Cdk 2, 4, and 6, as well as Cyclin D1, D2, D3, E1, and E2 knockout mice develop to adulthood [42]. Even compound knockout mouse embryos of Cdk2, 4, and 6 or compound knockouts of all D Cyclins exhibit normal organogenesis and survive up to 12 days post fertilization [19,21]. This indicates extreme resilience in the basic cell cycle machinery that drives the G1/S transition, challenging the classical dogma of sequential waves of Cdk4/6-Cyclin D and Cdk2-Cyclin E leading to normal S-phase entry [43]. Many of the genetic redundancies were recapitulated in our experiments by siRNA-mediated knockdown of Cyclins and Cdks in serum-starved RPE-1 cells, with the exception of strong dependence on Cyclin D1 and the combined action of Cdk4 and 6.

However, the most compelling finding presented here is that the cell cycle re-entry that follows a G1 arrest imposed by DNA damage is controlled differently than the cell cycle re-entry that follows serum starvation and contact inhibition. We show that S-phase entry after reversal of a DNA damage-induced checkpoint arrest in RPE-1 cells critically depends on full Cdk4/6 activity, for the inactivation of pocket proteins and inactivation of Cdh1. Depletion of Cdk4 or Cdk6 in RPE-1 cells diminishes the overall Cdk4/6 activity to such an extent that cell cycle re-entry after DNA damage is compromised. It is clear that this dependence on Cdk4/6 activity does not stem from defective DNA repair. While recovery requires Cdk4/6 kinase activity, it relies on more than the canonical inactivation of pocket proteins by Cdk-mediated phosphorylation. In this respect, we find recovery from a G1 arrest to be distinct from serum restimulation, as pocket protein inactivation by E7 oncoprotein is sufficient for S phase entry in serum-restimulated cells depleted of Cdk4 and −6, but not for S phase entry after irradiation. Using inducible expression of E7, we can further define this distinction with the observation that E7 expression before irradiation abolishes the G1 checkpoint, but E7 expression cannot reverse an established G1 arrest. Instead, both pocket protein and Cdh1 inactivation must be achieved to reverse the arrest imposed by DNA damage.

It is not unlikely that the dependency on Cdk4/6 we find here in damaged cells is related to the dependency on Cdk4 during tumorigenesis that was previously observed. Cdk4 is required for ErbB-2-driven mammary tumorigenesis, DMBA/TPA-induced skin tumor development, Men1^+/−^-driven neuroendocrine tumorigenesis, PDGF-induced glioma and APC^Min/+^-driven adenoma [44,45,46,47,48,49]. Conversely, Cdk4 and Cyclin D1 are frequently overexpressed in cancer, with *CCND1* amplification present in 20% of breast cancers and *CDK4* amplified in 18% of glioblastomas [50,51]. Thus, stimulation of early cell cycle progression through hyperactivation of Cyclin D/Cdk4 clearly presents a crucial growth benefit for cancer cells. We propose that on top of enhancing mitogenic signaling, such alterations also lower the threshold for proliferation in cells that have suffered damage to their DNA and may drive further mutation and carcinogenesis. Additionally, hyperactivation of Cyclin D/Cdk4 could help (early) tumor cells deal with ongoing replication stress that is frequently observed in cancer cells. Indeed, tumor resistant phenotypes of Cdk2 or Cdk4 knockout mice are lost in models that inactivate p53, arguing against a key role of mitogenic signaling per se [52]. As such, deregulation of Cdk4/6 and Cdh1 simultaneously could support growth factor independence and tolerance to DNA damage during tumorigenesis. As a consequence, inhibition of Cdk4/6 and/or activation of Cdh1 or pRb could provide a means to limit the proliferative capacity of tumor cells that have retained a functional G1 checkpoint.

## Data Availability

No new datasets were generated for this study. Data sharing is not applicable to this article.

## Supplementary Materials

The following are available online at https://www.mdpi.com/2073-4409/10/3/550/s1, Figure S1. (related to Figure 1); Figure S2 (related to Figure 4); Figure S3 (related to Figure 4 and Figure 5).
