# Supplementary material for: Combined Inactivation of Pocket Proteins and APC/CCdh1 by Cdk4/6 Controls Recovery from DNA Damage in G1 Phase
Source: Cells. 2021 Mar 4;10(3):550. doi: 10.3390/cells10030550 (PMC7999910; doi:10.3390/cells10030550)
Supplement: Supplementary file 1 [file cells-10-00550-s001.zip › FigS1.pdf]

Figure S1

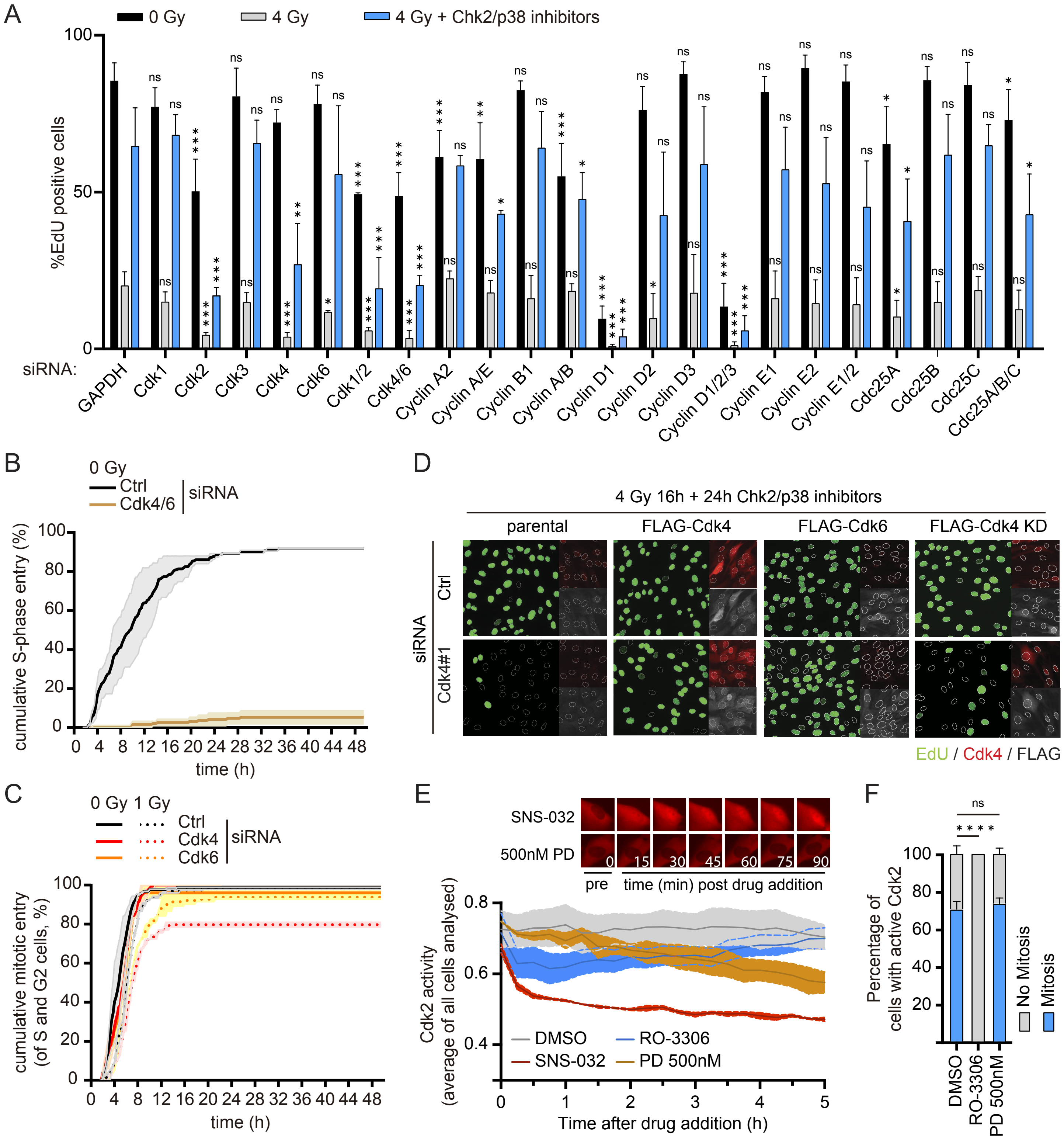

Figure S1. (related to Figure 1). (A) RPE-1 cells were transfected with the indicated siRNA during serum starvation and G1 recovery was assessed as in 1A. Depicted are the means and SD of 3 in-dependent experiments. Significance was calculated by comparing cells treated with different siRNAs to control-depleted cells subject to the same irradiation regimen (e.g., Cdk4-depleted 4 Gy irradiated cells were compared to control-depleted 4 Gy irradiated cells). **(B,C)** Asynchronously prolif-erating RPE-FUCCI cells were transfected with indicated siRNAs and irradiated (1 Gy) 24 to 48 h later. G1 cells (mKO2-hCdt1(30/120)+-mAG-hGem(1/110)-) in the starting frame were fol-lowed into S-phase (B), and G2 cells (mKO2-hCdt1(30/120)--mAG-hGem(1/110)+) were followed into mitosis (C). Depicted are the means and SEM of two independent experiments. **(D)** Recovery from 4 Gy induced by Chk2 and p38 inhibition in G1-synchronized RPE-1 cell lines expressing siRNA-resistant FLAG-Cdk4, FLAG-Cdk6 or kinase-dead (KD) FLAG-Cdk4. **(E)** RPE-1 cells sta-bly expressing a previously published Cdk2 activity reporter [34] were treated with a Cdk1 in-hibitor (RO-3306), a Cdk2 inhibi-tor (SNS-032) and the dual Cdk4/6 inhibitor PD0332991 for up to 5 h. Cdk2 activity was determined by calculating the average nucleo/cytoplasmic ratio of Cdk2-reporter fluorescence of at least 100 cells per condition per timepoint. Depicted are the means and SEM of three independent experiments. Inlay shows a cell before and after treatment with SNS-032 and 500 nM PD0332991, respectively. **(F)** Percentage of cells with active Cdk2 that enter into mitosis within 8 h after they were treated as in (E). Depicted are means and SEM of three independent experiments. For all panels, significance was calculated using a one-sided un-paired *t*-test. * $p < 0.05$, ** $p < 0.01$, *** $p < 0.005$, **** $p < 0.0001$, ns = not significant.
