# Supplementary material for: Combined Inactivation of Pocket Proteins and APC/CCdh1 by Cdk4/6 Controls Recovery from DNA Damage in G1 Phase
Source: Cells. 2021 Mar 4;10(3):550. doi: 10.3390/cells10030550 (PMC7999910; doi:10.3390/cells10030550)
Supplement: Supplementary file 1 [file cells-10-00550-s001.zip › FigS2.pdf]

Figure S2

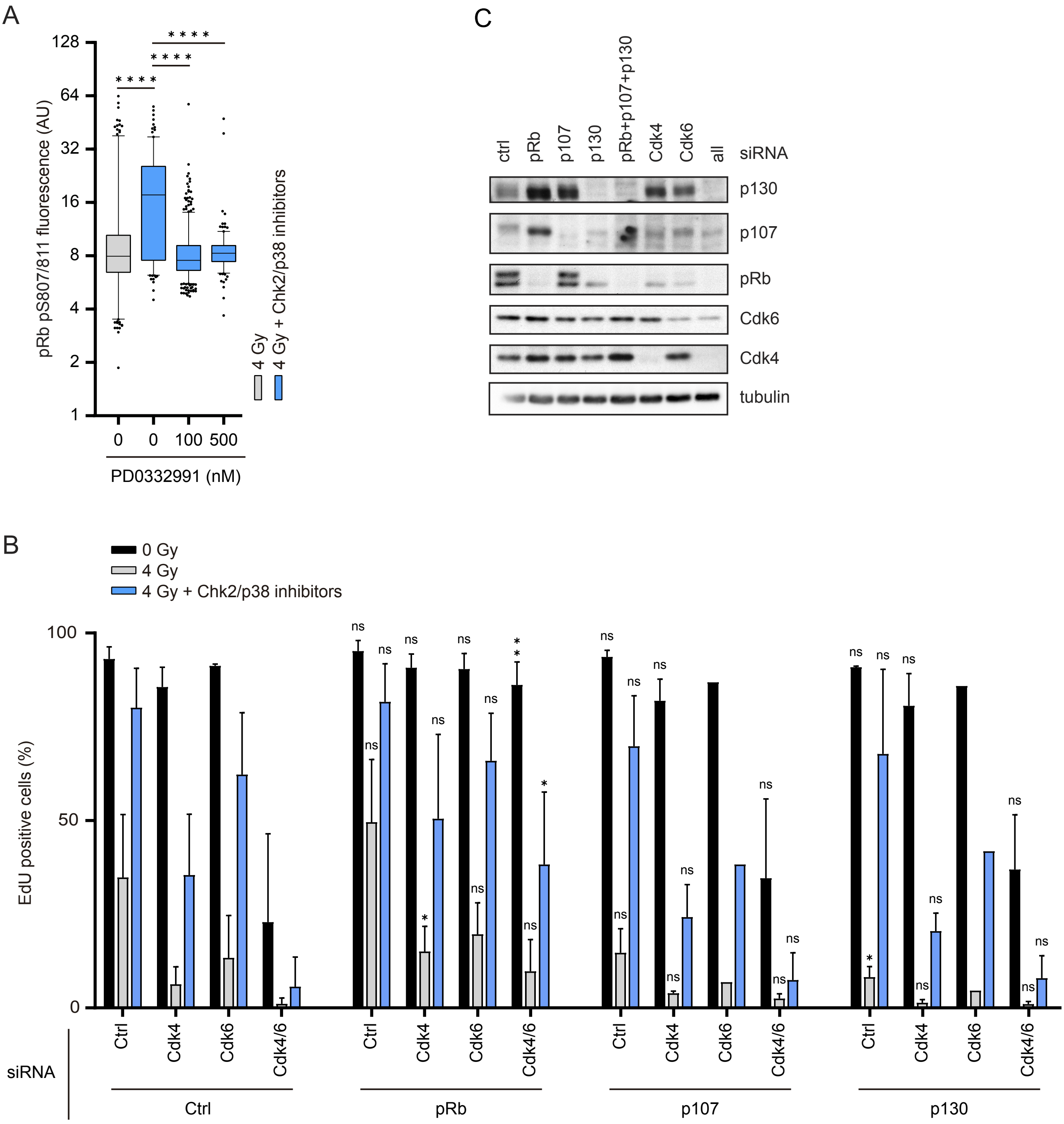

Figure S2 (related to Figure 4). (A) RPE-1 cells synchronized in G1 were irradiated (4 Gy). 16 h post irradiation Chk2/p38 inhibitors were added alone or together with the indicated concentra-tions of PD0332991 for 8 h. Cells were then fixed for immunofluorescence detection of S807/811-phosphorylated pRb. **(B)** RPE-1 cells were transfected with the indicated siRNA during serum starvation and G1 recovery was assessed as in Figure 1A. Depicted are the means and SD of two to five independent experiments. Significance was calculated by comparing the knock-down of different Cdks in combination with either pRb, p107 or p130 knockdown to the different Cdk knock-downs combined with a control siRNA, treated with a similar irradiation regimen (e.g., pRb+Cdk4-depleted 4 Gy irradiated samples are compared to con-trol+Cdk4-depleted 4 Gy irradiated samples). **(C)** Cells treated as in B were harvested for West-ern Blot. For all panels, significance was calculat-ed using a one-sided unpaired *t*-test. * $p < 0.05$, ** $p < 0.01$, *** $p < 0.005$, **** $p < 0.0001$, ns = not significant.
