# Supplementary material for: Combined Inactivation of Pocket Proteins and APC/CCdh1 by Cdk4/6 Controls Recovery from DNA Damage in G1 Phase
Source: Cells. 2021 Mar 4;10(3):550. doi: 10.3390/cells10030550 (PMC7999910; doi:10.3390/cells10030550)
Supplement: Supplementary file 1 [file cells-10-00550-s001.zip › FigS3.pdf]

Figure S3

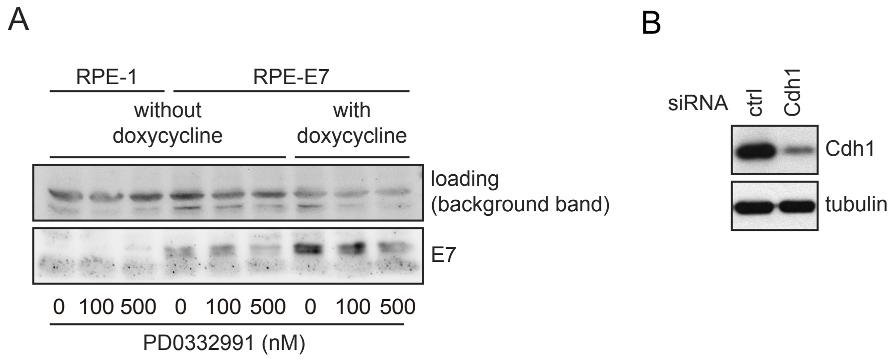

Figure S3 (related to Figures 4 and 5). (A) Wild type RPE-1 and RPE-E7 cells were grown in the presence of the indicated drugs for 24 h, and subsequently harvested for Western Blot analysis of E7 expression. **(B)** RPE-1 cells were transfected with control or Cdh1-targeting siRNAs and har-vested for Western Blot 48 h later.
